# Pathological post-systolic shortening as a prognostic marker for major cardiovascular events in patients with type 2 diabetes

**DOI:** 10.1186/s44156-025-00085-0

**Published:** 2025-09-01

**Authors:** Lina Hult, David Kylhammar, Jan Engvall, Carl Johan Östgren, Fredrik Nyström, Peter Blomstrand, Kristofer Hedman

**Affiliations:** 1 Department of Clinical Physiology and, Department of Health, Medicine and Caring Sciences, Linköping University, Linköping, Sweden; 2 Wallenberg Centre for Molecular Medicine, Linköping University, Linköping, Sweden; 3 CMIV, Center of Medical Image Science and Visualization, Linköping University, Linköping, Sweden; 4 Department of Health, Medicine and Caring Sciences, Linköping University, Linköping, Sweden; 5 Department of Natural Science and Biomedicine, School of Health and Welfare, Jönköping University, Jönköping, Sweden

**Keywords:** Diabetes mellitus, 2D Echocardiography, Post-systolic shortening, Global longitudinal strain, Prognostics, Adverse cardiovascular outcome

## Abstract

**Background:**

Post-systolic shortening (PSS) has emerged as a method for evaluating left ventricular dysfunction. We aimed to determine whether pathological PSS, alone or in combination with global longitudinal strain (GLS), is a prognostic factor for major adverse cardiovascular events (MACEs) in patients with type 2 diabetes. We prospectively investigated 364 patients with type 2 diabetes aged 55–65 years in the CARDIPP study. All patients underwent echocardiography between 2005 and 2009. PSS, measured by speckle tracking echocardiography, was defined as myocardial contraction after aortic valve closure. Pathological PSS was defined as a post-systolic index > 5% and was calculated as follows: [(maximum longitudinal strain – peak systolic longitudinal strain)/(maximum longitudinal strain)]. The endpoint was any MACE, defined as hospitalization or death due to heart failure, myocardial infarction, or stroke. Cox proportional hazard ratios (HR) with 95% confidence intervals (CI) were calculated and adjusted for sex, age, body mass index, hypertension, smoking, previous cardiovascular events, and HbA1c level. The mean follow-up time was 11.2 ± 2.3 years.

**Results:**

Pathological PSS was associated with an increased risk of MACEs after adjustment for other cardiovascular risk factors (HR 2.20, 95% CI 1.11–4.37). Subjects with reduced GLS, PSS and GLS combined in a risk prediction model, had an adjusted HR for MACEs of 2.94 (95% CI 1.33–6.52).

**Conclusions:**

Our results suggest that PSS may provide additional prognostic information for patients with T2D when used alone or in combination with GLS.

**Supplementary Information:**

The online version contains supplementary material available at 10.1186/s44156-025-00085-0.

## Background

Cardiovascular disease (CVD) remains the leading cause of death worldwide, although it is largely preventable [1]. Patients with type 2 diabetes (T2D) are at greater risk of developing CVD compared to the general population, mainly because of the elevated risk of atherosclerosis [2–4]. Identifying individuals with T2D who are at a particularly high risk of CVD or incipient heart failure is important for customizing and intensifying medical and lifestyle interventions that could delay or even prevent CVD progression [5].

**Fig. 1:**
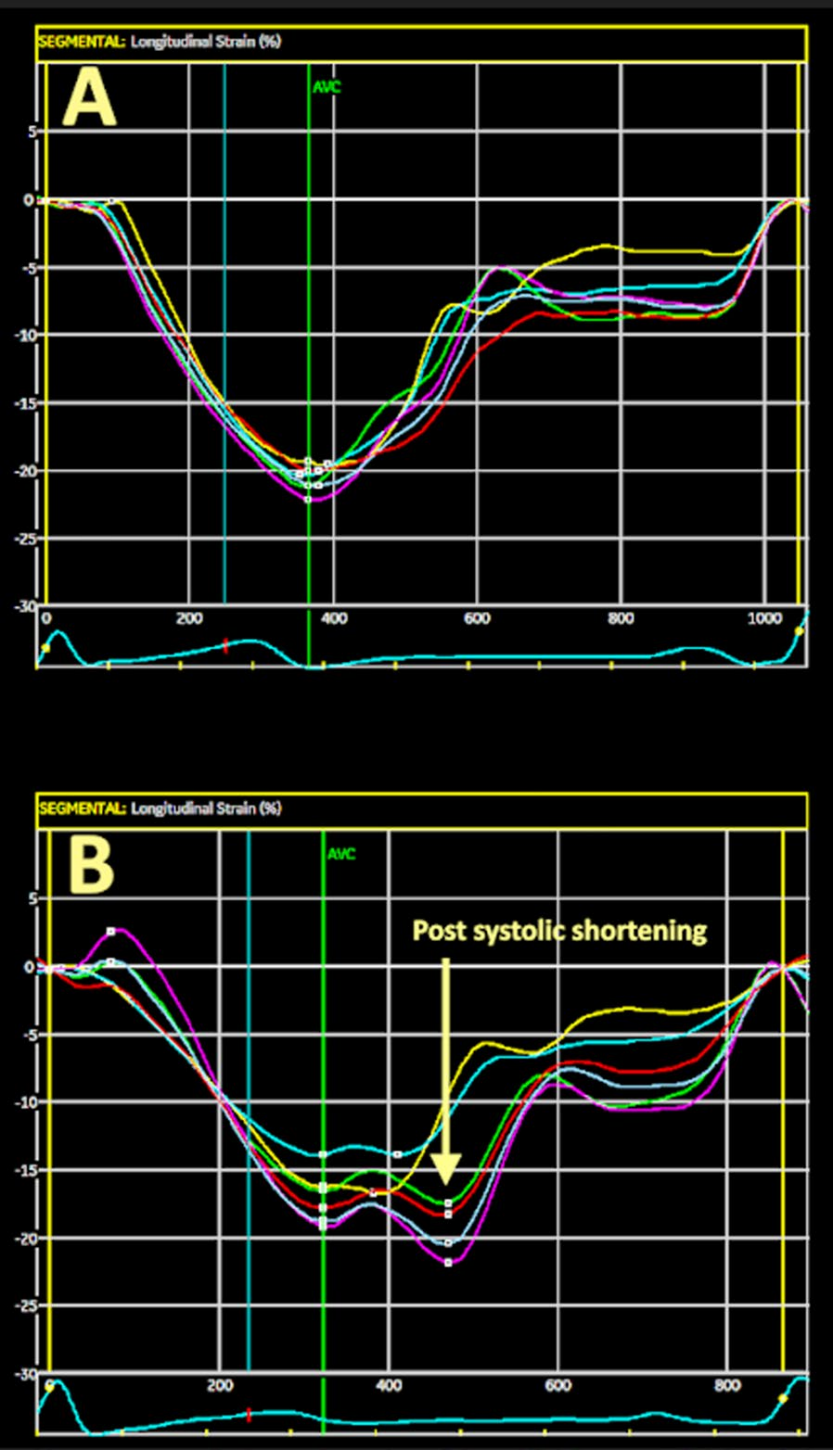
Screen capture of longitudinal strain profiles. AVC, aortic valve closure. **A** shows a normal strain curve with its maximum at the end of systole. **B** shows a strain curve with pathological post-systolic shortening, where peak strain was reached after AVC in several segments

In addition to traditional risk evaluation for CVD, variations in echocardiographic profiling have shown promising results in identifying patients at high risk for cardiovascular events [3, 6]. Strain curves derived from speckle tracking echocardiography (STE) are of special interest because they enable detailed analysis of ventricular function and identify small alterations in wall deformation that are not visible to the naked eye and undetectable by standard measures of ventricular function, such as left ventricular ejection fraction (LVEF) [7]. One measurement that can be derived from STE curve analysis is the presence and extent of post-systolic shortening (PSS) (Fig. 1).

PSS, defined as myocardial shortening that continues after aortic valve closure (AVC), has demonstrated promising results in predicting cardiovascular events in the general population. However, as of our current knowledge, its efficacy in yielding similar results in individuals with diabetes remains largely unverified [8].

Our objectives were to investigate, in a population of patients with T2D but with relatively few other risk factors for CVD: (1) the degree to which PSS was present; (2) the relationship between PSS and other echocardiographic markers of systolic dysfunction, such as LVEF and global longitudinal strain (GLS); and (3) whether PSS can predict future major adverse cardiovascular events (MACEs) in this group.

## Methods

### Study population

Patients with T2D were recruited for the CARDIPP study (CArdiovascular Risk factors in patients with DIabetes: a prospective study in primary care [ClicialTrials.gov number NCT 01049737]). The study protocol has been previously described in detail [3, 9]. In brief, patients with T2D, aged 55–65 years, were invited by specialized nurses at 13 primary healthcare centres in the county of Östergötland, Sweden, between May 2005 and January 2009.

Patients with severe valvular pathology and/or severe physical illness with a life expectancy of < 1 year or with severe mental illness that impaired their ability to provide informed consent were excluded. A total of 535 participants were recruited into the CARDIPP study, and data on their medical history and current medical therapies were registered. Systolic and diastolic blood pressures were measured with the patient in a sitting position, and the mean of three measurements was used. Venous blood samples were collected for glucose, lipid, and blood count analysis. Anthropometric data, electrocardiograms, and a thorough medical history were obtained. Body mass index (BMI) was calculated as body weight (kg)/body length (m ^2^). BSA was calculated according to the DuBois and DuBois formula [10].

In the current study, relying on high-quality strain curves, we additionally excluded patients with a pacemaker, atrial fibrillation, left or right bundle branch block, or ≥ 2 left ventricular segments missing from the STE analysis. Study inclusion is depicted in supplementary material figure S1 and number of segments per individual available for analysis supplementary table S1.

The study's primary endpoint was the composite of MACEs, defined as hospitalization for or death from heart failure, myocardial infarction (MI), or stroke, with the time to the first diagnosis of any event recorded. Outcome data were sourced from the Swedish National Cause-of-Death Registry and the National Patient Register, which logs all hospital visits and diagnoses. Participants were followed from study inclusion to December 31, 2018, or death.

### Echocardiography

Comprehensive echocardiography was performed by trained and experienced sonographers using GE Vivid 7 ultrasound systems (GE Vingmed Ultrasound, General Electric, Chicago, IL, USA). Apical views (four-chamber, two-chamber, and apical long-axis views) were recorded at a frame rate > 40 frames/s.

The longitudinal strain was analysed offline using 2D STE (Q-analysis in EchoPAC version 203, GE Healthcare, Chicago Illinois, USA) by an experienced examiner with > 5 years of experience in strain analyis. Myocardial tracking was automatically outlined, followed by visual assessment, and manual adjustment when needed [11, 12]. If the tracking failed or parts of the myocardial wall were obscured, the affected segment was excluded from the analysis. Aortic valve closure was automatically identified by the software and visually verified in the apical long axis view and manually corrected when necessary. GLS was calculated as the average of maximum strain measurements from 18 left ventricular segments according to recommendations from the European Association of Cardiovascular Imaging [13].

LVEF was measured using the modified Simpson method. Mitral inflow patterns were measured at the tip of the mitral leaflets using pulsed Doppler imaging and the maximum velocity of the E-wave was measured. Tissue Doppler measurements were performed at the septal and lateral mitral annuli in the four-chamber view, and the peak of the early diastolic velocity (e′) was evaluated. E/e′ was used as a measurement of diastolic dysfunction, e′ was the mean value from the septal and lateral walls, and a value of E/e′ > 14 was considered elevated [14]. LVEF < 50% and GLS less than − 16% were considered reduced [13, 15]. Inter- and intra-variability was calculated by means of intraclass correlation coefficient (ICC). We randomly selected 20 individuals, and the ICC was calculated for matching pairs.

### Post-systolic shortening

**Fig. 2:**
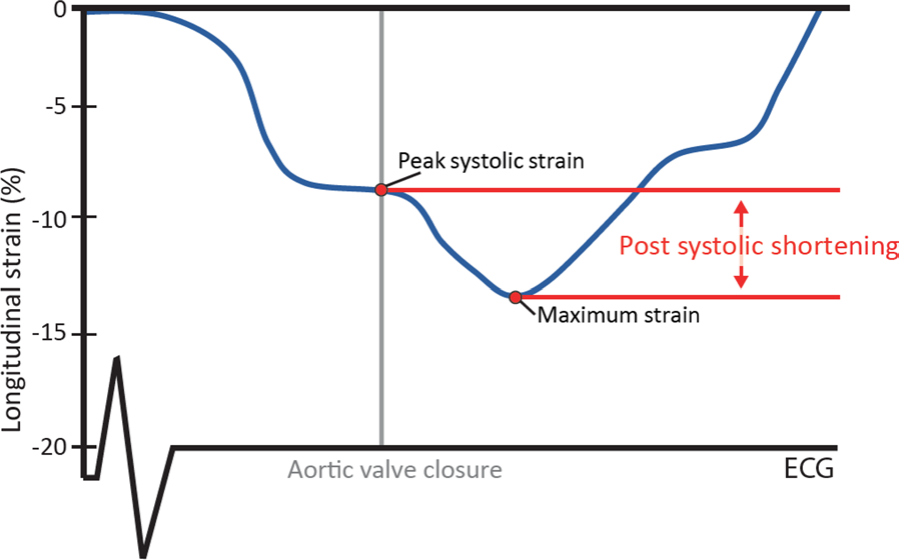
Schematic illustration of a longitudinal strain curve and the definition of post-systolic shortening. AVC, aortic valve closure; ECG, electrocardiography

Maximum global and peak systolic longitudinal strain were determined for each left ventricular segment, where PSS was defined as the difference between the peak systolic longitudinal strain and maximum longitudinal strain (Fig. 2).

For quantification of PSS, a post-systolic index (PSI) for all segments was calculated as.

$$[(maximumlongitudinal \, strain \, {-}peak \, systolic \, longitudinal \, strain) \, / \, \left( {maximum \, longitudinal \, strain} \right)]\,$$

The arithmetic mean of the PSI for all segments was calculated and used in the analyses. Previous studies have shown that some form of PSS is present in 35% of the general population and in 31% of healthy individuals [8, 16]. To exclude those with PSS within the normal range we chose a cut-off value of PSI above 5% to denote pathological PSS consistent with a previous study of normal values in the general population [17].

### Statistical analysis

All statistical analyses were performed using SPSS version 29 (SPSS Inc., Chicago, IL, USA). Categorical variables are presented as absolute numbers (percentages), and continuous variables as means (± standard deviation). Group comparisons were conducted using appropriate statistical tests, including the independent samples t-test, Mann–Whitney U-test, or chi-square test, depending on the type and distribution of data. Statistical significance was set at P < 0.05.

Survival rates were determined using the Kaplan–Meier method and compared using the log-rank test. Univariate and multivariate Cox proportional hazard models were used to calculate hazard ratios (HRs) and confidence intervals (CIs) to evaluate the prognostic value of pathological PSS. The analyses were adjusted stepwise as follows: in Model 1, for sex and age; Model 2 was additionally adjusted for previous cardiovascular events, history of heart failure, MI, stroke, percutaneous coronary intervention (PCI), coronary artery bypass graft (CABG), hypertension, smoking, diabetes duration, BMI, and HbA1c; Model 3 was adjusted as model 2 and for E/e′.

For sub-analysis, the cohort was subdivided into four groups based on GLS and the presence of pathological PSS: (1) normal GLS and normal PSS; (2) normal GLS and abnormal PSS; (3) abnormal GLS and normal PSS; and (4) abnormal GLS and abnormal PSS.

## Results

### The presence and extent of pathological PSS

364 met the inclusion criteria and had an image quality that permitted analysis of PSS and the subsequent calculation of PSI. A comparison between included and excluded participants is shown in supplementary table S2. The groups were similar in clinical characteristics, except for a significantly higher BMI among excluded individuals. Baseline patient characteristics are shown in Table 1. For baseline characteristics between men and women see table S3 in supplementary material.

**Table 1:** Baseline characteristics

Baseline characteristics	All n = 364
Demographic data	
Age, years	60.6 (± 3.1)
Female	117 (32%)
Body mass index, kg/m^2^	29.5 (± 4.4)
Body mass index > 30 kg/m^2^	149 (40)
Waist circumference, cm	102.6 (± 11.9)
Clinical characteristics	
Hear Rate	66 (± 11)
Diabetes duration, years	6.6 (± 5.5)
Heart failure diagnosis	4 (1)
Previous coronary revascularisation	25 (7)
Angina pectoris	24 (7)
Previous stroke	9 (3)
Previous myocardial infarction	26 (7)
Hypertension diagnosis	237 (66)
Systolic blood pressure, mmHg	136 (± 15)
Diastolic blood pressure, mmHg	81 (± 10)
Smoking	73 (20)
Medication	
Loop diuretics	19 (5)
Statins	214 (59)
ACEI and/or ARB	174 (48)
Calcium channel blocker	61 (17)
β-Blocker	130 (36)
ASA	120 (33)
Oral diabetic medication	200 (55)
Insulin	97 (27)
Blood chemistry	
HbA1c, %	6.9 (± 1.0)
Triglycerides, mmol/L	1.7 (± 0.9)
Cholesterol, mmol/L	4.7 (± 1.0)
LDL-C, mmol/L	2.6 (± 0.7)
Creatinine, mmol/L	91 (± 16)
GFR, mL/min/1.73 m^2^	71 (± 12)
Echocardiographic characteristics	
LVEF, %	53 (± 8)
GLS, %	− 17.2 (± 2.5)
PSI, %	3.2 (± 3.2)
E/e′, ratio, unit-less	12.6 (± 4.0)
LVEDV, mL	93 (± 24)
LVEDVI, mL/m^2^	46 (± 10)
LVESV, mL	45 (± 21)
LVESVI, mL/m^2^	22 (± 11)

Continuous variables were expressed as mean ± standard deviation, and categorical variables were expressed as absolute numbers (percent)

ACEI,  angiotensin-converting enzyme inhibitor; ARB,  angiotensin receptor blocker; ASA,  acetylsalicylic acid; HbA1c,  glycosylated haemoglobin; LDL, low-density lipoprotein; GFR,  glomerular filtration rate; LVEF,  left ventricular ejection fraction; GLS,  global longitudinal strain; PSI,  post-systolic index; E/e′; E,  transmitral E-wave velocity, e′,  early diastolic mitral annulus velocity; LVEDV,  left ventricular end-diastolic volume; LVEDVI,  left ventricular end-diastolic volume index; LVESV,  left ventricular end-systolic volume; LVESVI,  left ventricular end-systolic volume index

The mean intra-observer variability for average measures of GLS yielded an ICC of 0.95 (0.85–0.98), while for PSS the ICC was 0.80 (0.95–5.03). The mean inter-observer variability for average measures resulted in an ICC (95% CI) of 0.93 (0.76–0.98) for GLS and 0.88 (0.97–8.28) for PSS.

At inclusion, 62 (17.0%) individuals presented with pathological PSS (i.e., PSI > 5%), of which 16 (25.8%) had previously experienced an MI or had been revascularised with either CABG or PCI.

In total, 36 (9.9%) individuals had either experienced MI or undergone revascularisation with either PCI or CABG at the time of inclusion. These 36 individuals were considered to have pre-existing CVD, and out of these, 16 (44.4%) had pathological PSS. Among those with no known CVD, 46 (14.0%) had pathological PSS at the time of inclusion. Thus, pathological PSS was more prevalent in subjects with pre-existing CVD than in those without CVD at study inclusion (44.4% vs. 14.0%, p < 0.001).

**Fig. 3:**
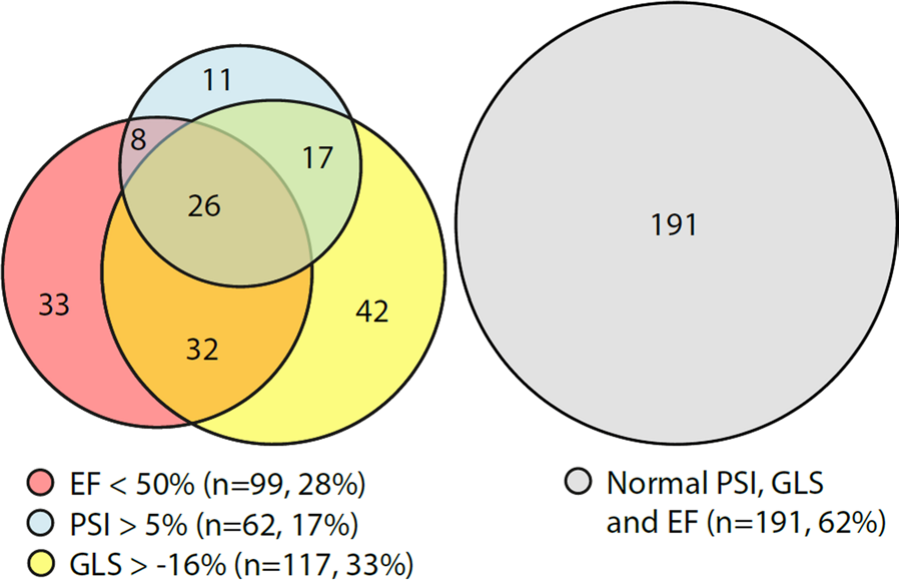
A Venn diagram showing the relationship between reduced LVEF, reduced GLS, and pathological PSS (i.e. PSI > 5%) in the cohort. Note that several individuals with preserved EF (≥ 50%) still exhibited abnormal PSI or GLS. These individuals are represented within the yellow and blue circles but outside the red EF < 50% circle. LVEF, left ventricular ejection fraction; GLS, global longitudinal strain; PSS, post-systolic shortening

The relationships between reduced LVEF, GLS, and pathological PSS in the entire cohort are shown in Fig. 3. As illustrated 62% of subjects exhibited normal values for all systolic function markers. Among those with impaired systolic function, a reduction in GLS (< 16%) was the most common, followed by a decrease in LVEF (< 50%), whereas pathological PSI (> 5%) was least common. Only 11 (18%) subjects had isolated PSI elevation. The most significant overlap occurred between LVEF and GLS, with approximately 50% exhibiting reductions in both parameters.

### PSS and cardiovascular outcome

A total of 46 participants (12.6%) experienced a MACE during follow-up, with 55 events recorded in total (some individuals experienced more than one event).

Compared to those with no or normal PSS, participants with pathological PSS had a higher unadjusted risk of MACEs (HR 3.73. 95% CI 2.06–6.76), which persisted after adjustment for sex, age, history of cardiovascular disease, hypertension, current smoking, diabetes duration, BMI, and HbA1c (HR 2.20. 95% CI 1.11–4.37). See also Kaplan Meier curves presented in supplement material figure S2. A reduced LV function in terms of GLS less than -16% or LVEF < 50% was also associated with higher unadjusted risk, although statistical significance did not persist after adjustment (Table 2).

**Table 2:** The risk of MACEs associated with exceeding/falling below the threshold value for four different echocardiographic variables

	Unadjusted	Model 1	Model 2	Model 3
Adjusted for	–	Age, sex	As Model 1 + cardiovascular disease^a^, diabetes duration (years), HbA1c, BMI, current smoking, hypertension	As Model 2 + E/e′
PSI > 5 (vs. ≤ 5)	**3.73** (2.06–6.76)	**3.83** (2.11–6.95)	**2.20** (1.11–4.37)	1.76 (0.84–3.67)
GLS > − 16% (vs. ≤ − 16%)	**2.61** (1.46–4.67)	**2.78** (1.55–4.99)	1.87 (0.97–3.61)	1.71 (0.88–3.34)
LVEF < 50% (vs. ≥ 50%)	**1.85** (1.03–3.36)	**1.94** (1.08–3.54)	1.65 (0.86–3.18)	1.65 (0.86–3.16)

PSI,  post-systolic index. GLS,  global longitudinal strain. LVEF,  left ventricular ejection fraction. HbA1c,  glycosylated haemoglobin. BMI,  body mass index

^a^History of stroke, angina pectoris, or ischaemic heart disease

After additional adjustment for E/e′, no measure of systolic function was statistically significant in predicting MACEs. For the results of the univariate Cox regression analysis for each risk factor refer to supplementary table S4. When analysing PSI as a continuous variable, the unadjusted HR for MACEs was 1.16 (CI 1.09–1.22) and the adjusted HR was 1.11 (CI 1.04–1.19) per percentage-point increase in PSI. When excluding all individuals with a history of ischemic heart disease (i.e., prior myocardial infarction and revascularization procedures like CABG or PCI) we were left with 327 individuals. In this group, 34 (10%) individuals experienced a MACE (myocardial infarction, congestive heart failure, or stroke) during the follow-up period. When analyzing PSI as a (continuous variable) within this subgroup, the unadjusted HR for MACE was 1.09 (CI 1.01–1.18) per percentage point increase in PSI (p = 0.036).

### Combining PSS and GLS for risk prediction

Among those with both normal GLS and normal PSI 92% (n = 226) experienced no events, while 8% (n = 18) experienced one or more MACEs. In the group with abnormal GLS but normal PSI 87% (n = 76) had no events, and 13% (n = 10) experienced at least one event. For patients with normal GLS but pathological PSI 84% (n = 19) had no events, and 16% (n = 3) experienced one or more events. In the group with both abnormal GLS and pathological PSI 65% (n = 43) had no events, and 35% (n = 15) experienced at least one event (p < 0.001).

**Fig. 4:**
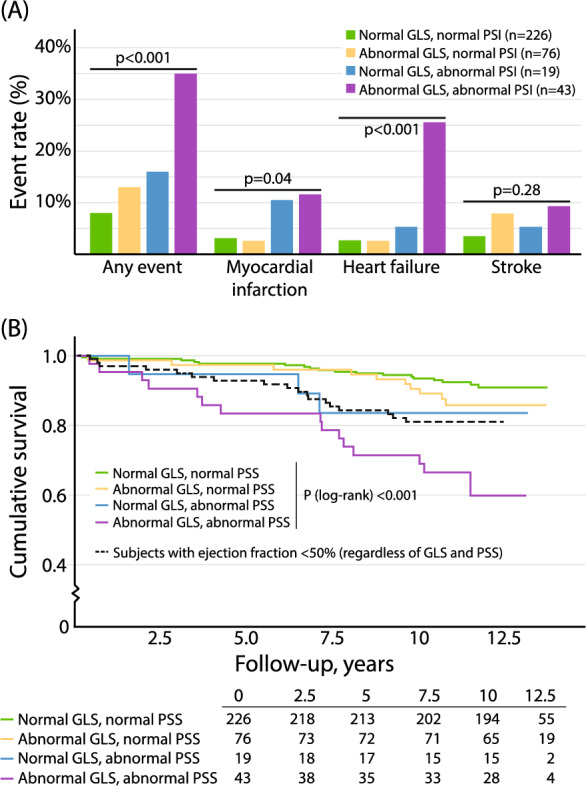
**A** Percentage of events for study participants with either normal or pathological PSS and GLS values. Pathological PSS was defined as a PSI of > 5%. A GLS of less than − 16% was considered reduced. It should be noted that, in this analysis, a single participant could experience more than one event. **B** Survival rates of study participants with either normal or pathological PSS and GLS and for information survival rates of study participants with reduced LVEF regardless of PSS and GLS. PSS, post-systolic shortening; PSI, post-systolic index; GLS, global longitudinal strain; LVEF, left ventricular ejection fraction

Regarding specific outcomes, myocardial infarction occurred in 3.1% (n = 7) of patients with normal GLS/normal PSI, 2.6% (n = 2) with abnormal GLS/pathological PSI, 11% (n = 2) with normal GLS/pathological PSI, and 11.6% (n = 5) with abnormal GLS/pathological PSI (p = 0.003). Heart failure was observed in 2.7% (n = 6) of those with normal GLS/normal PSI, 2.6% (n = 2) with abnormal GLS/pathological PSI, 5.3% (n = 1) with normal GLS/pathological PSI, and 25.6% (n = 11) with abnormal GLS/pathological PSI (p < 0.001). Stroke rates did not significantly differ across the groups, with rates ranging from 3.5% to 9.3% (p = 0.28) (Fig. 4, see also supplementary material’s table S5).

When combining PSS and GLS in a risk prediction model with different combinations of normal and abnormal PSS and GLS, the only combination that was significantly associated with MACEs was GLS less than -16% together with PSI > 5% (Table 3). Statistical significance persisted after adjustment for sex, age, previous cardiovascular disease (stroke, angina pectoris, heart failure, or ischaemic heart disease), diabetes duration, HbA1c, BMI, current smoking, hypertension, and E/e′.

**Table 3:** The risk of major adverse cardiovascular events associated with different combinations of normal and pathological PSS and GLS

	Unadjusted	Model 1	Model 2	Model 3
Adjusted for	–	Age, sex	As Model 1 + cardiovascular disease^a^, diabetes duration (years), HbA1c, BMI, current smoking, hypertension	As Model 2 + E/e′
PSI ≤ 5% and GLS ≤ − 16%	Reference	Reference	Reference	Reference
GLS > − 16% and PSI ≤ 5%	1.66 (0.77–3.60)	1.78 (0.82–3.86)	1.29 (0.56–2.99)	1.35 (0.58–3.12)
PSI > 5% and GLS ≤ − 16%	2.23 (0.66–7.58)	2.30 (0.68–7.83)	1.16 (0.26–5.16)	1.07 (0.24–4.78)
GLS > − 16% and PSI > 5%	**5.38** **(2.71–10.71)**	**5.66** **(2.84–11.28)**	**2.94** **(1.33–6.52)**	**2.34** **(1.00–5.51)**

The reference group is those with normal PSS and GLS. The bold font indicates a statistically significant hazard ratio

PSI,  Post-Systolic Index; GLS,  Global Longitudinal Strain; HbA1c,  glycosylated haemoglobin; BMI,  Body Mass Index

^a^History of stroke, angina pectoris, heart failure, or ischaemic heart disease

## Discussion

The main finding of this observational prospective study of middle-aged patients with T2D was that pathological PSS is a predictor of MACEs, and when combined with reduced GLS, risk prediction improves even further.

Although, PSS does not contribute to the ejection of blood [18], prior research has found that PSS is present to some degree in healthy individuals and has been suggested to reflect ventricular reshaping during the isovolumetric relaxation time [16]. A more pronounced PSS is considered pathological and has been observed in acutely ischaemic and scarred myocardium to a degree corresponding to the extent of myocardial injury [16]. To account for the possibility of non-pathological PSS, we opted to use an indexation approach, setting a cut-off value of 5%, which aligns both with our own data and the threshold suggested by Brainin et al. [17].

The cause of pathological PSS has been debated, and both active and passive contractions have been suggested as possible mechanisms [19]. Claus et al. showed in a mathematical model and in simulation studies that PSS most likely occurs in damaged myocardium as a passive motion resulting from pulling tension from neighbouring normal, or near-normal myocardium, and that the ability to produce PSS suggests that the affected myocardium still has preserved elasticity and is not fibrotic [20]. Other studies have found that in patients with acute ischaemia PSS could predict recovery of systolic function, suggesting that PSS, in that setting, is a marker of viability [21, 22]. PSS has also been shown to be a reliable marker of ischaemia during dobutamine stress echocardiography [23, 24].

Previous studies have mainly focused on PSS in acute ischemia; however, Brainin et al. [8] reported PSS as a significant predictor of cardiovascular events in the general population and later in a subset of T2D patients [8, 25]. In the present study, we could show that these results were robust in a T2D population with longer follow up time. We defined pathological PSS as PSI > 5% [17]. However, it should be noted that it may be appropriate to use different cut-off values in subjects with manifest CVD, acute ischaemia, and myocardial scarring [16]. At baseline 17% of our cohort exhibited pathological PSS, notably more common in subjects with prior MI, PCI, CABG, or heart failure, as anticipated [26].

At baseline, abnormal GLS, calculated from maximum systolic strain in the cardiac cycle emerged as the most frequent indicator of systolic dysfunction, followed by LVEF. GLS, highly sensitive, detects early subclinical myocardial dysfunction, while LVEF, although associated with higher mortality in severely reduced cases, weakly predicts mortality in mildly reduced or near-normal cases making it unsuitable as a predictor for CVD [7, 27–29]. Although pathological PSS was the least common marker of systolic dysfunction, some individuals exhibited only pathological PSS. This suggests that PSS may offer unique insights into left ventricular systolic dysfunction, not captured solely by LVEF and GLS analyses.

In this study pathological PSS was equally potent as reduced GLS in predicting our endpoint of MACEs even after adjusting for established cardiovascular comorbidities. We also showed that the combination of abnormal GLS and pathological PSS was more accurate in predicting MACEs than the respective systolic markers analysed alone, suggesting an additional effect of the combination of GLS and PSS. However, since abnormalities in these parameters sometimes occurred independently, evaluating them separately remains valuable. This supports the combined use of GLS and PSS, as PSS alone may miss a significant subgroup.

The cohort was relatively well medicated, with lipid and HbA1c and blood pressure levels within an acceptable range or only slightly elevated. This suggests that despite T2D, their cardiovascular risk was only moderately increased, and this was confirmed by the relatively few MACEs during follow-up. Other studies with similar populations have reported a higher incidence of MACEs [30, 31]. Studies from the general population with participants of a similar age range have shown a higher incidence of comparable MACEs than Cardipp T2D [8].

Even in this relatively low risk setting, pathological PSS was a significant predictor of MACEs, and especially for MI and heart failure. When individuals with a history of ischemic cardiovascular disease were excluded, the association between PSI and MACE remained significant. This indicates that post-systolic shortening is of value also in subjects with no manifest ischemic heart disease.

It should be noted that we excluded participants with atrial fibrillation, left or right bundle branch block, or pacemaker rhythm from the analyses, as they can be expected to have a high incidence of PSS, irrespective of cardiovascular risk.

Most post systolic shortening occurs during early diastole in the isovolumetric relaxation time when LV pressure drops quickly [17, 32]. This timing could possibly influence LV relaxation and make it dependent on diastolic dysfunction. Previous studies have shown that E/e′ is a strong marker of adverse cardiovascular events in individuals with T2D which might contribute to the loss of significance of PSI when adjusting for E/e*.* However, when pathological PSS was combined with reduced GLS, the risk prediction of MACEs remained significant even after adjusting for E/e′, again suggesting that the combination of PSS and GLS is a powerful marker of CVD.

Although the clinical application of speckle-tracking–based parameters such as PSS and GLS remains to be fully established, our findings may contribute to the ongoing effort to refine cardiovascular risk stratification in patients with T2D. With rapid advances in artificial intelligence and automated echocardiographic analysis, parameters like PSS and GLS could potentially be incorporated into AI-driven models. Such models may allow identification of high-risk subgroups, and our study can therefore be seen as a hypothesis-generating contribution to this evolving field.

### Limitations

Image quality is important when analysing STE and considering that the image material for this study was gathered more than a decade ago, it was necessary to exclude some echocardiographic examinations because of poor quality, which is a limitation. However, it was still possible to analyse most individuals.

The relatively low number of events limits the generalizability of our findings. Although the cohort size is moderate, the number of patients experiencing MACE was rather small, and the results should therefore be interpreted with some caution. Future studies with higher event rates and longer follow-up are needed to validate these observations.

There were relatively few cardiovascular events despite the long follow-up period, which could be due to the voluntary nature of the study; thus, the healthy participant effect cannot be excluded. We did not apply formal correction for multiple testing, as our analyses were based on predefined hypotheses and a limited number of clinically relevant variables; however, we acknowledge the potential risk of type I error. Due to the low rate of other cardiovascular risk factors, we included patients with prior CVD and heart failure in the analysis. Additionally, undetected subclinical coronary artery disease (CAD) may have influenced the observations related to post-systolic shortening. Furthermore, data on regional wall motion abnormalities, which could also contribute to PSS were not available and should be considered a potential limitation.

Our analysis was based on time to first event; we did not evaluate the total number of events or apply competing risk methods, which may have led to underestimation of recurrent or mutually exclusive outcomes.

There are several variations in how to analyse PSS, we chose to quantify it through indexation and used a mean value from all segments in the STE analysis. Currently, there is no gold standard which is a limit to its clinical applicability, and further research is needed to establish the optimal method.

## Conclusion

Our results suggest that pathological PSS, defined by a PSI > 5%, may provide prognostic information regarding the risk of MACE in patients with T2D. When PSS is combined with GLS, risk prediction appears stronger. However, given the low number of events, these findings require validation in larger cohorts.

## Supplementary Information

Supplementary file 1.

## Data Availability

The dataset supporting the conclusions of this article cannot be shared online due to privacy restrictions but will be shared upon reasonable request from the corresponding author.

## Abbreviations

AVC: Aortic valve closure
BMI: Body Mass Index
CABG: Coronary Artery Bypass Graft
CI: Confidence interval
CVD: Cardiovascular disease
GLS: Global longitudinal strain
HR: Hazard Ratios
ICC: Intraclass correlation coefficient
LVEF: Left ventricular ejection fraction
MACE: Major adverse cardiovascular event
MI: Myocardial infarction
PCI: Percutaneous coronary intervention
PSI: Post-Systolic Index
PSS: Post-systolic shortening
STE: Speckle tracking echocardiography
T2D: Type 2 diabetes
