# Supplementary material for: Pathological post-systolic shortening as a prognostic marker for major cardiovascular events in patients with type 2 diabetes
Source: Echo Res Pract. 2025 Sep 1;12:21. doi: 10.1186/s44156-025-00085-0 (PMC12400764; doi:10.1186/s44156-025-00085-0)
Supplement: Supplementary file 1 [file 44156_2025_85_MOESM1_ESM.docx]

**Pathological Post Systolic Shortening: A Prognostic Marker for Major Cardiovascular Events in Patients with Type 2 Diabetes**

**Supplement material**

**Supplement Figure S1.** Flowchart depicting inclusion in the study.

**
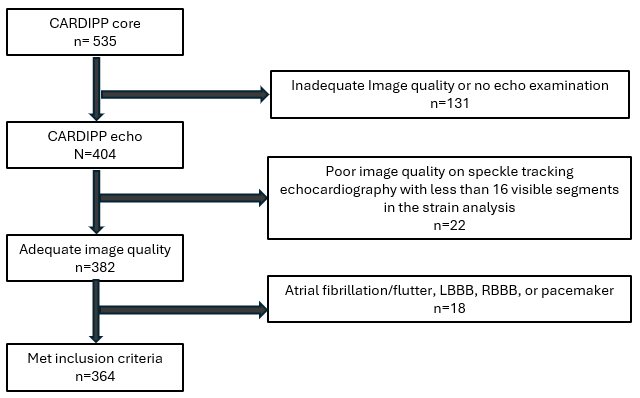
**

**Supplement table S1.** Number of segments per individual available för analysis.

| **Number of segments:** | **N = 364 individuals** | **%** |
| --- | --- | --- |
| **18** | **231** | **63,5** |
| **17** | **94** | **25,8** |
| **16** | **39** | **10,7** |

**Supplement table S1.** Note that individuals with < 16 segments were excluded from analyses from the start and are not included in this table.

**Supplement table S2.** Comparison of baseline characteristics between included and excluded participants.

|  | **Included n=364** | **Excuded n=171** | **p-value** |
| --- | --- | --- | --- |
| Age, years | 60 (±3.1) | 60 (±3.0) | 0.929 |
| Female | 117 (32) | 55 (32) | 0.996 |
| Body mass index, kg/m2 | 29.5 (4.4) | 31.9 (±5.2) | <0.001 |
| Diabetes duration, years | 6.6 (±5.5) | 7.0 (±5.5) | 0.432 |
| Hypertension diagnosis | 237 (66) | 112 (66) | 0.954 |
| History of CVD * | 36 (10) | 20 (12) | 0.517 |
| Smoking | 73 (20) | 32 (18.9) | 0.762 |

**Supplement table S2**. Comparison of baseline characteristics between included and excluded participants. *=History of myocardial infarction or CABG/PCI. Continuous variables were expressed as mean ± standard deviation, and categorical variables were expressed as absolute numbers (percent).

**Supplement table S3.** Baseline characteristics for men and women.

| **Baseline Characteristics** | **Women**  **n=117 (32%)** | **Men**  **n=247 (68%)** |
| --- | --- | --- |
| **Demographic Data** |  |  |
| Age, years | 60.7 (±3.0) | 60.6 (±3.1) |
| Body mass index, kg/m^2^ | 30.4 (±5.4) | 29.0 (±3.7) |
| Body mass index >30 kg/m² | 57 (49) | 92 (37) |
| Waist circumference, cm | 101.1 (±14.5) | 103.3 (±10.4) |
| **Clinical Characteristics** |  |  |
| Heart Rate | 68 (±12) | 65 (±11) |
| Diabetes duration, years | 7.5 (±5.9) | 6.2 (±5.3) |
| Heart failure diagnosis | 0 (0) | 4 (2) |
| Previous coronary revascularisation | 3 (3) | 22 (9) |
| Angina pectoris | 5 (4) | 19 (8) |
| Previous stroke | 3 (3) | 6 (2) |
| Previous myocardial infarction | 5 (4) | 21 (9) |
| Hypertension diagnosis | 72 (63) | 165 (68) |
| Systolic blood pressure, mmHg | 138 (±16) | 136 (±15) |
| Diastolic blood pressure, mmHg | 80 (±10) | 82 (±10) |
| Smoking | 30 (26) | 43 (17) |
| **Medication** |  |  |
| Loop diuretics | 9 (8) | 10 (4) |
| Statins | 71 (61) | 143 (58) |
| ACEI and/or ARB | 55 (47) | 119 (48) |
| Calcium channel blocker | 17 (14) | 44 (18) |
| β-Blocker | 38 (33) | 92 (37) |
| ASA | 39 (33) | 81 (33) |
| Oral diabetic medication | 62 (53) | 138 (56) |
| Insulin | 35 (30) | 62 (25) |
| **Blood Chemistry** |  |  |
| HbA1c, % | 6.7 (±1.0) | 6.9 (±1.0) |
| Triglycerides, mmol/L | 1.7 (±0.9) | 1.7 (±0.9) |
| Cholesterol, mmol/L | 5.0 (±1.0) | 4.5 (±1.0) |
| LDL-C, mmol/L | 2.7 (±0.8) | 2.6 (±0.7) |
| Creatinine, mmol/L | 77 (±15) | 91 (±16) |
| GFR, mL/min/1.73 m^2^ | 69 (±13) | 72 (±12) |
| **Echocardiographic Characteristics** |  |  |
| LVEF, % | 53 (±7) | 53 (±8) |
| GLS, % | -17.4 (±2.4) | -17.1 (±2.4) |
| PSI, % | 3.3 (±3.2) | 3.1 (±3.2) |
| E/e’, ratio, unit-less | 13.8 (±4.0) | 12.0 (±3.9) |
| LVEDV, mL | 79 (±17) | 100 (±24) |
| LVEDVI, mL/m² | 42 (±8) | 48 (±10) |
| LVESV, mL | 37 (±11) | 48 (±24) |
| LVESVI, mL/m² | 20 (±6) | 23 (±12) |
| LV Mass index, g/m² | 110 (±23) | 126 (±28) |
| La Volume Index, mL/m² | 21 (±2) | 20 (±2) |

**Supplement Table S3.** Baseline Characteristics. Continuous variables were expressed as mean ± standard deviation, and categorical variables were expressed as absolute numbers (percent). ACEI = angiotensin-converting enzyme inhibitor. ARB = angiotensin receptor blocker. ASA = acetylsalicylic acid. HbA1c = glycosylated haemoglobin. LDL, low-density lipoprotein. GFR = glomerular filtration rate. LVEF = left ventricular ejection fraction. GLS = global longitudinal strain. PSI = post-systolic index. E/e’; E= transmitral E-wave velocity, e’= early diastolic mitral annulus velocity. LVEDV = left ventricular end-diastolic volume. LVEDVI = left ventricular end-diastolic volume index. LVESV = left ventricular end-systolic volume. LVESVI = left ventricular end-systolic volume index. LV = left ventricular. La = Left atrial.

**Supplement Figure S2.** Kaplan Meier for the whole cohort.


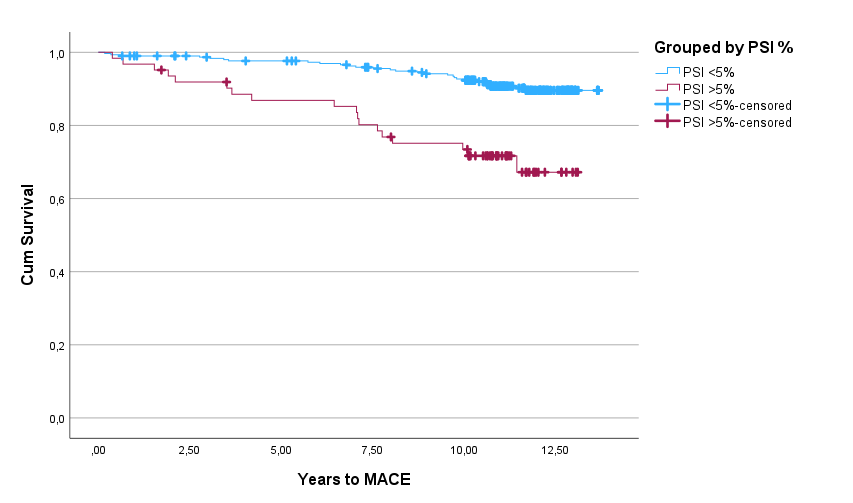


**P = <0.001**

**Supplement Figure S2**. Kaplan Meier for the whole cohort. Kaplan Meier for the whole cohort. Endpoints: myocardial infarction, heart failure and/or stroke, hospitalization, or death in. The blue line represents those with PSI < 5 % and the red line those with PSI > 5 %. PSI = post systolic index.

**Supplement table S4:** Univariate Cox proportional hazard analysis to examine the association of different variables with MACE as an outcome.

|  | HR | 95% CI | P-value |
| --- | --- | --- | --- |
| Male sex | 1.28 | 0.62-2.65 | 0.5 |
| Age | 1.13 | 1.02-1.26 | 0.02 |
| Previous Cardiovascular Disease* | 3.25 | 1.56-6.79 | 0.02 |
| Hypertension diagnosis | 2.78 | 1.27-6.09 | 0.01 |
| Current smoking | 2.66 | 1.35-5.22 | 0.005 |
| Diabetes duration | 1.00 | 0.95-1.06 | 0.92 |
| BMI | 1.11 | 1.04-1.19 | 0.03 |
| HbA1c DCCT | 1.30 | 0.99-1.70 | 0.06 |
| PSI > 5 % | 2.20 | 1.11-4.37 | 0.02 |

**Supplement table S4**. Univariate Cox proportional hazard analysis to examine the association of different variables with MACE as an outcome. * = angina, heart failure, myocardial infarction, coronary artery bypass graft, percutaneous intervention, or stroke. BMI = Body Mass Index.

**Supplement table S5:** Frequency of events in groups based on normal/abnormal GLS and PSI

|  | Normal GLS, Normal PSI  (n=226) | Abnormal GLS, normal PSI (n=76) | Normal GLS, pathological PSI (n=19) | Abnormal GLS, pathological PSI (n=43) | Chi2 |
| --- | --- | --- | --- | --- | --- |
| No event | 208 (92%) | 66 (87%) | 16 (84%) | 28 (65%) | p<0.001 |
| ≥1 event* | 18 (8%) | 10 (13%) | 3 (16%) | 15 (35%) |  |
| Myocardial Infarction (I21) | 7 (3.1%) | 2 (2.6%) | 2 (11.0%) | 5 (11.6%) | 0.003 |
| Heart failure (I50) | 6 (2.7%) | 2 (2.6%) | 1 (5.3%) | 11 (25.6%) | <0.001 |
| Stroke (I60-I63) | 8 (3.5%) | 6 (7.9%) | 1 (5.3%) | 4 (9.3%) | 0.28 |
| *) Note that an individual can experience multiple events during follow-up. | | | | | |

**Supplement table S5.** Frequency of events in groups based on normal/abnormal GLS and PSI. This table is represented in the article by the diagram in figure 4 and in the manuscript text. PSI = post systolic index. GLS = global longitudinal strain.
